# Left Ventricular Strain Is Associated With Myocardial Recovery Following ST-Elevation Myocardial Infarction, a Prospective Longitudinal CMR Study

**DOI:** 10.3389/fcvm.2022.842619

**Published:** 2022-02-24

**Authors:** Mohamad B. Taha, Eric I. Jeng, Michael Salerno, Diego Moguillansky, Ellen C. Keeley, Mohammad A. Al-Ani

**Affiliations:** ^1^Department of Cardiovascular Medicine, DeBakey Heart and Vascular Center, Houston Methodist Hospital, Houston, TX, United States; ^2^Department of Surgery, Division of Thoracic and Cardiovascular Surgery, College of Medicine, University of Florida, Gainesville, FL, United States; ^3^Department of Medicine, Division of Cardiology, University of Virginia, Charlottesville, VA, United States; ^4^Department of Medicine, Division of Cardiology, College of Medicine, University of Florida, Gainesville, FL, United States

**Keywords:** STEMI, CMR, FT-CMR, infarct size, LV global strains

## Abstract

**Background:**

Infarct size following ST-elevation myocardial infarction (STEMI) is an important determinate of left ventricular (LV) dysfunction and cardiovascular morbidity and mortality. Cardiac magnetic resonance feature tracking (CMR-FT) is a technique that allows for the assessment of myocardial function via quantification of longitudinal, radial, and circumferential strain. We investigated the association between CMR-FT-derived myocardial global strain and myocardial recovery.

**Methods:**

A prospective study on patients presenting with STEMI treated with primary percutaneous coronary intervention (PCI) was conducted. CMR imaging was obtained at two interval time points, the baseline within 2 weeks of hospital discharge and follow-up at 6 months. Strain analysis was performed via FT-CMR, and recovery was quantified by the area of late gadolinium enhancement (LGE).

**Results:**

A total of *n* = 14 patients met inclusion and exclusion criteria and were analyzed. There was a significant reduction in the infarct size, as measured by LGE mass percentage of the left ventricular muscle mass, between the initial and follow-up CMR (19.7%, IQR 12.2–23.9 vs. 17.1%, IQR 8.3–22.5, *p* = 0.04). Initial strain parameters were inversely correlated with the initial edema mass and the decrease in LGE mass between the initial and follow-up CMR. All LV global strains had high accuracy for the prediction of a reduction in LGE mass by 50% or more.

**Conclusions:**

LV global strains measured after primary PCI can predict the extent of myocardial recovery.

## Introduction

Infarct size following ST-elevation myocardial infarction (STEMI) is a major determinate of left ventricular (LV) dysfunction and clinical outcomes. The risk of recurrent cardiovascular events, morbidity, and mortality following STEMI has remained considerable, despite marked diagnostic and medical advances. Thus, the reliable assessment of myocardial injury post-STEMI is clinically relevant to the management of heart failure and myocardial recovery. Cardiac magnetic resonance (CMR) can assess infarct size and quantify scar by late gadolinium enhancement (LGE), myocardial edema, salvaged myocardium, and microvascular obstruction (MVO), all of which provide crucial prognostic information (1–4).

Myocardial strain analysis is a measure of contractile function that is relatively independent of myocardial loading conditions (5). The recent development of CMR feature-tracking (CMR-FT) techniques allows performing longitudinal, radial, and circumferential strain in a more precise manner compared to echocardiography (6, 7). Direct assessment of strain has been shown to be of high prognostic value to systolic function in STEMI patients (8–10). We hypothesized that FT-CMR-derived myocardial global strain correlates with the degree of myocardial recovery in STEMI patients after successful primary percutaneous coronary intervention (PCI).

## Materials and Methods

### Study Participants and Protocol

We enrolled a cohort of patients presenting with STEMI treated with primary PCI between August 2014 and August 2019. The study was carried out in concordance with the principles of the Declaration of Helsinki and was approved by the university institutional review board. All patients provided written informed consent. Inclusion criteria for the study were age >18 years, presentation to the emergency room with STEMI based on universal electrocardiogram (ECG) criteria (11), and clinical management with primary PCI. Exclusion criteria were (1) history of prior acute coronary syndrome; (2) renal insufficiency with glomerular filtration rate <45 ml/min/1.73 m^2^; (3) contrast allergy; and (4) any contra-indication to CMR.

### Cardiac Magnetic Resonance (CMR) Imaging Acquisition and Analysis

All patients underwent baseline CMR imaging within 2 weeks of discharge and follow-up at 6 months. Imaging was ECG-gated and was conducted on a 1.5-Tesla scanner. The standardized imaging protocol was obtained based on the society of cardiovascular magnetic resonance guidelines for imaging post-myocardial infarction (12). A stack of short-axis steady-state free precession cine images covering the left ventricle (LV) from apex to base was obtained with the following sequence parameters: repetition time (TR) 2.7 ms, echo time (TE) 1.3 ms, flip angle 73°, field of view (FOV) 300–350 mm and resolution 1.8 × 1.4 × 8.0 mm. LGE images were obtained 10–15 min after injection of 0.15 mmol/kg contrast agent gadoterate meglumone (Dotarem; Guebert, France) using a phase-sensitive inversion recovery pulse sequence with the following sequence parameters: TR 7.1 ms, TE 3.4 ms, flip angle 25°, FOV 300–340 mm, and resolution 1.8 × 1.3 × 8 mm. T2 mapping was obtained via T2-prepared steady state free precession readout acquisitions at effective echo times of 0, 24, and 55 ms per slice. Quality control maps (*R*^2^) were used to rule out artifact prior to analysis.

**Figure 1:**
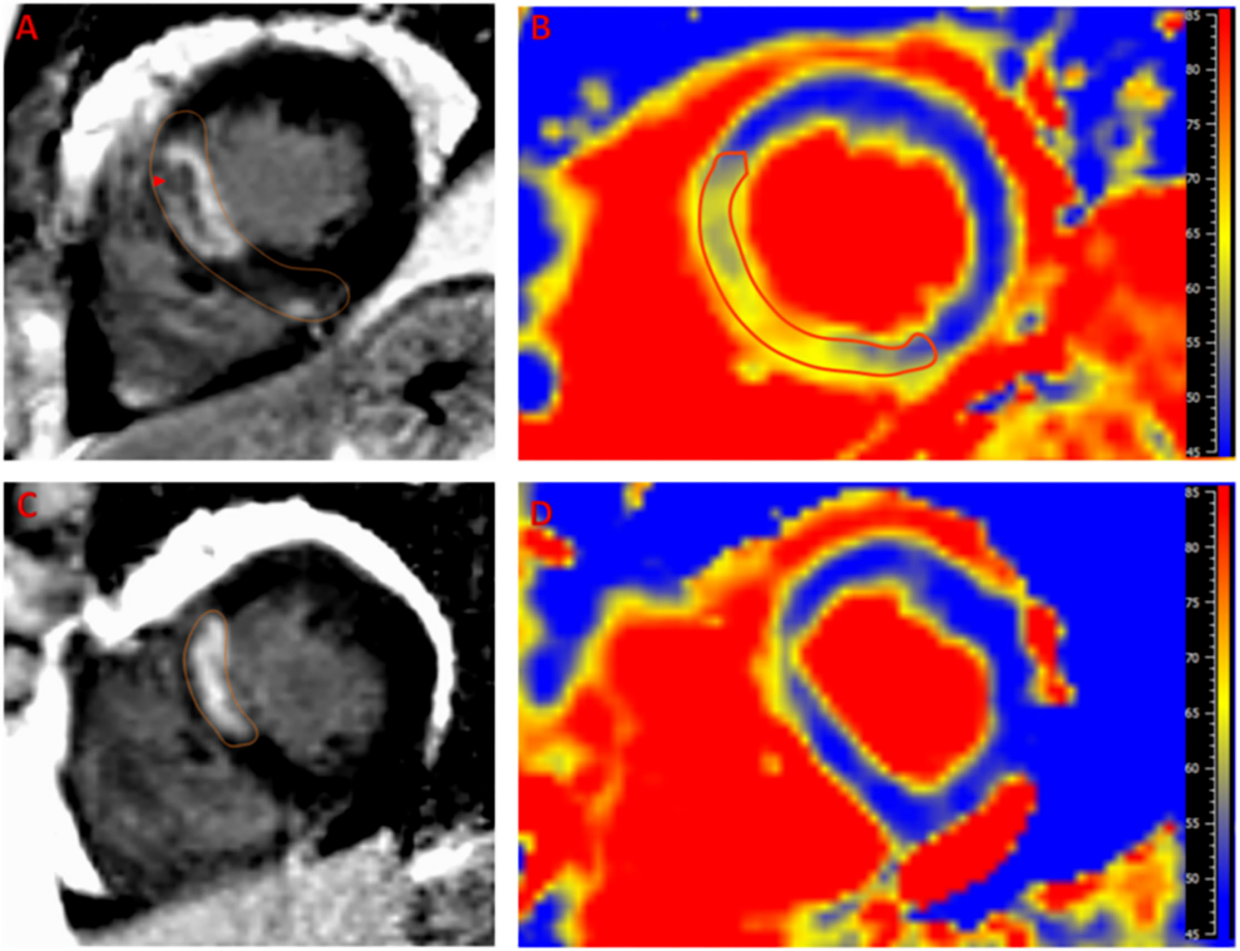
Example of a case of left anterior descending artery infarction. Late gadolinium enhancement (LGE) is noted in the septal segments **(A)** with area microvascular obstruction (arrow head). Edema (yellow hue) as detected by T2 mapping **(B)** extends beyond the LGE area with notable decreased T2 time in the microvascular obstruction region indicating hemorrhage. The 6-month follow up scan shows marked regression of LGE **(C)** and edema **(D)** with residual infarction in the anteroseptum that appears thinned on T2 map with minimal residual T2 elevation that is likely related to fibrous tissue deposition. Reference T2 time for normal myocardium in our laboratory is <55 ms.

Images were analyzed by a CMR-certified cardiologist using Medis software (Medis Suite 3.0, Netherlands). For assessment of cardiac function on initial images, end-diastolic and end-systolic endocardial and epicardial cavity areas were semi-automatically delineated for each short-axis and long-axis slice with care taken to exclude papillary muscles and trabeculae from the endocardial contours. Total LV mass, end-diastolic and systolic volume, stroke volume, and cardiac output were calculated by standard methods and indexed for body surface area. For assessment of myocardial fibrosis on LGE images, endocardial and epicardial LV borders of hyperenhanced scar tissue were semi-automatically delineated for each short-axis slice at the site of infarction and manually corrected. Areas of LGE and edema were defined in comparison to remote areas using the full width half maximum method (Figure 1). Manual corrections were applied to exclude blood pool and trabeculations. In order to avoid volume averaging artifacts, 10% margins from the endocardium and epicardium were excluded. MVO was defined as a central dark focus within the LGE area. Myocardial salvage index (MSI) was calculated as a percentage of infarction mass (inferred by LGE mass) from the total edema mass (inferred by high T2 mass).

### Strain Analysis

**Figure 2:**
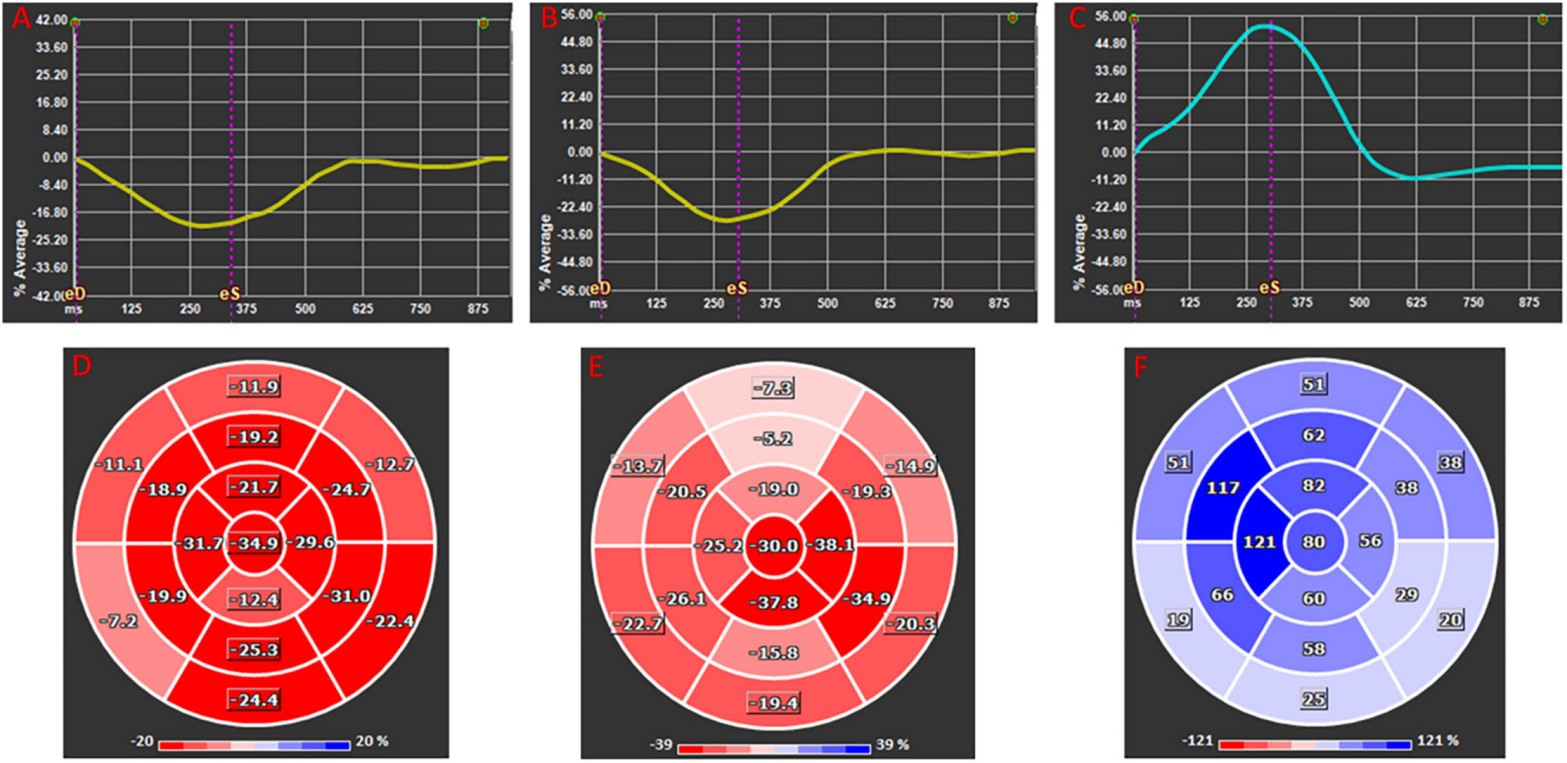
Global longitudinal **(A,D)**, circumferential **(B,E)**, and radial **(C,F)** 2D strain presented as average global strain curves **(A–C)** and corresponding segmental strain according to American Heart Association 17 segments Bull's eye ventricular maps **(D–F)**. Note that longitudinal and circumferential strains are negative at their peak as they represent decrease in in fiber dimension whereas radial strain is positive as it infers increased myocardial thickness.

Two-dimensional CMR-FT strain analysis was performed using dedicated and validated software (Medis Suite 3.0, Netherlands). Myocardial, epicardial, and endocardial contours were traced using the semi automated method. Papillary muscles were excluded. End systole and end-diastole were defined in reference to aortic and mitral valve closure times, respectively. The standard 17-segment model was applied in the analysis of LV longitudinal strain measured in two-, three-, and four-chamber views. The basal, mid-cavity, and apical levels were segmented from the end-diastolic four-chamber long-axis cine image. For circumferential and radial strain analysis of LV short-axis views, a modified 16-segment model (omitting the apical cap) was applied, using the RV insertion point as the reference point for the junction of LV anterior wall and septum. For short-axis strain analysis, the base was selected as the slice still showing a complete circumference of myocardium throughout the entire cardiac cycle (without through-plane distortion from the LV outflow tract), and the apex was selected as the slice still showing LV cavity at systole. Global longitudinal, radial, and circumferential strain values (GLS, GRS, and GCS) were automatically extracted from corresponding strain curves in 2D mode (Figure 2).

### Statistical Analysis

Continuous data were presented as median with interquartile range (IQR), and categorical variables were presented as numbers with corresponding percentages. Differences in continuous and categorical variables between two groups were tested by *t*-test and χ^2^ test, respectively. Correlations were assessed using Pearson's correlation coefficient (*r*) and linear regression model between continuous variables.

The discriminative power of IS predictors was quantified by receiver operating characteristic (ROC) analyses. We obtained area under the curve (AUC) values by the non-parametric DeLong method. AUC values were categorized as negligible ( ≤ 0.55), small (0.56–0.63), moderate (0.64–0.70), and strong (≥0.71). The optimal cutoff values for dichotomization of continuous IS predictors were identified by the Youden Index. For all tests, a two-tailed *P* < 0.05 was considered statistically significant. Statistical analysis was conducted using GraphPad Prism 9.0.2 for Windows (GraphPad Software, La Jolla California, USA).

## Results

### Study Population

A total of 14 patients completed both baseline and 6-month follow-up CMR and were included in the analysis. Baseline clinical characteristics are provided in Table 1. Culprit lesion involved the left anterior descending (LAD) artery in 9 (64%), the right coronary artery (RCA) in 4 (29%), and the left circumflex artery (LCX) in 1 (7%) of patients. Patients received evidence-based medical therapy (13). Three patients developed cardiogenic shock, and one was treated with an intra-aortic balloon pump. All patients survived the hospital course and were able to undergo follow-up CMR examinations.

**Table 1:** Baseline clinical characteristics.

**Baseline characteristics**	**STEMI patients**
Sample size, *n*	14
Age, median (IQR)	53.5 (50.5–56.8)
Female, *n* (%)	3 (21)
**Comorbidities**
Hypertension, *n* (%)	5 (36)
Diabetes mellitus, *n* (%)	3 (21)
Hyperlipidemia, *n* (%)	7 (50)
Smoker, *n* (%)	11 (79)
Chronic kidney disease, *n* (%)	1 (7)
**Laboratory findings**
Peak cardiac troponin pg/mL, median (IQR)	66.4 (36–82.7)
Admission creatinine mg/dL, median (IQR)	1 (0.8–1.0)
**Culprit lesion**, ***n*** **(%)**
LAD	9 (64%)
LCX	1 (7%)
RCA	4 (29%)
**Number of affected vessels**, ***n*** **(%)**
1	9 (64%)
2	4 (29%)
3	1 (7%)
Number of LV segments involved, median (IQR)	5.5 (5.0–7.0)
**Medical therapy**
Dual anti-platelet therapy, *n* (%)	14 (100)
Beta-blocker, *n* (%)	11 (79)
ACEi/ARB, *n* (%)	13 (93)
Statin, *n* (%)	14 (100)
**Outcomes**
90-day hospitalization, *n* (%)	0
90-day mortality, *n* (%)	0

*ACEi/ARB, angiotensin-converting enzyme inhibitor/angiotensin II receptor antagonists; IQR, inter-quartile range; LAD, left anterior descending artery; LCX, left circumflex artery; RCA, right coronary artery*.

### CMR Parameters

Initial CMR scan was performed within a median of 10 days (IQR, 2.5–18), and the follow-up CMR scan was performed 6 months (IQR, 6–6.5) after STEMI. An overview of CMR parameters for the initial and follow-up CMR are summarized in Table 2. For initial CMR-FT strain parameters, median LV GLS was −13.5% (IQR, −16.6 to −12.3), GRS was 43.8% (IQR, 38.4–47.8), and GCS was −17.8% (IQR, −21.8 to −14).

**Table 2:** CMR findings.

**CMR parameters^£^**	**Initial CMR**	**Follow-up CMR**	***P*-value**
LV EDV, mL	137.8 (111.4–177.5)	147.5 (108.2–156.3)	0.38
LV EDVI, mL/m^2^	74.7 (62.9–89.8)	72.6 (60–87.4)	0.41
LV ESV, mL	83.2 (42.8–104.8)	85.2 (50.5–98.3)	0.35
LV ESVI, mL/m^2^	45.2 (28.7–58.3)	44.2 (25.3–47.7)	0.26
LV EF, %	46.3 (37.7–63.2)	49.2 (31.6–63.3)	0.77
LV SV, mL	72.8 (61.6–78.8)	49.2 (43.4–83)	0.20
CO, L/min	4.1 (3.5–5)	3.6 (2.8–5.6)	0.68
CI, L/min/m^2^	1.9 (1.7–2.4)	1.6 (1.4–2.4)	0.50
LVM, g	118.9 (111.4–142.9)	121.8 (97.8–148.3)	0.52
LVMI, g/m^2^	60.6 (57.0–68.5)	65.4 (49.5–70.7)	0.67
LGE mass, % of LVM	19.7 (12.2–23.9)	17.1 (8.3–22.5)	0.04*
Edema mass, % of LVM	31.1 (21.2–35)	—	—
MSI	0.2 (0.2–0.4)	—	—
MVO mass, % of LGE mass	8.5 (7.1–11.7)	8.5 (2.5–13.0)	0.12
RV EDV, mL	144.8 (135.5–154.1)	134.8 (124.4–145.1)	0.41
RV EDVI, mL/m^2^	72.2 (67.9–76.4)	67 (62.9–71.0)	0.36
RV EF, %	54 (52.1–55.9)	53 (51.8–54.5)	0.58
LV GLS, %	−13.5 (−16.8 to −12.2)	−15.5 (−17.8 to −14.9)	0.10
LV GRS, %	43 (38.4–47.8)	43.1 (34.0–59.4)	0.68
LV GCS, %	−17.8 (−21.8 to −13.9)	−19.5 (−21.7 to −14.3)	0.98

CMR, cardiac magnetic resonance; IQR, inter-quartile range; LV EF, left ventricular ejection fraction; LV GCS, left ventricular global circumferential strain; LV GLS, left ventricular global longitudinal strain; LV GRS, left ventricular global radial strain; LVM, left ventricular mass; LVMI, left ventricular mass index; LV EDV, left ventricular end-diastolic volume; LV EDVI, left ventricular end-diastolic volume index; LV ESV, left ventricular end-systolic volume; LV ESV, left ventricular end-systolic volume index; LV SV, left ventricular stroke volume; LGE, late gadolinium enhancement; MVO, microvascular obstruction; MSI, myocardial salvageable index; CO, cardiac output; CI, cardiac index; RV EDV, right ventricular end-diastolic volume; RV ESVI, right ventricular end-systolic volume index; RV EF, right ventricular ejection fraction. *P <0.05;^£^CMR parameters data are presented as median with interquartile range (IQR).

**Figure 3:**
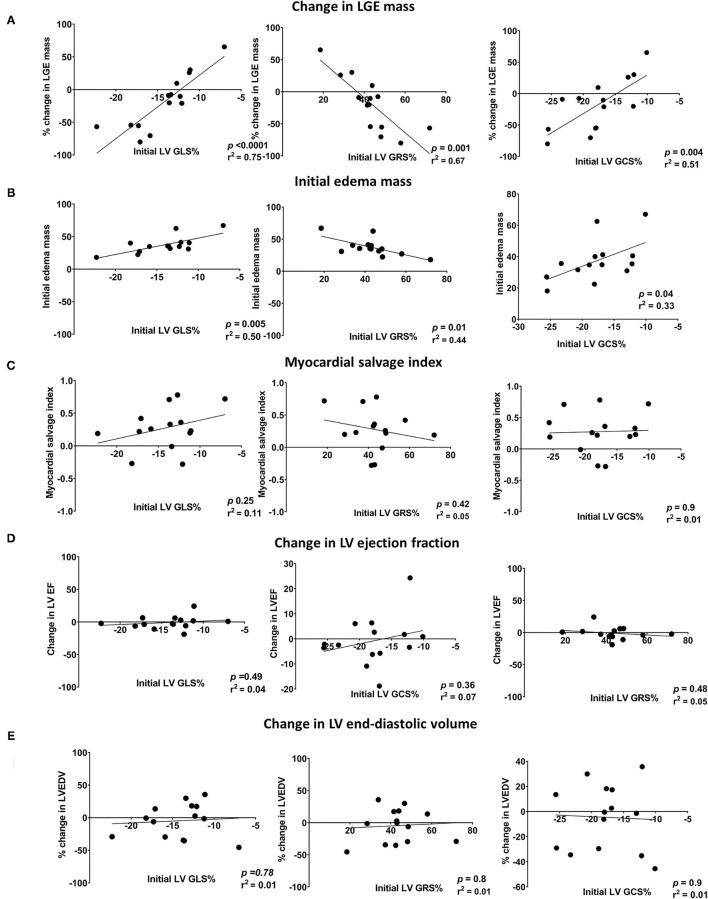
Scatter plots showing the linear correlations between initial LV global strains (GLS, GRS, and GCS) and changes in **(A)** LGE mass, **(B)** edema mass, **(C)** LVEF, **(D)** CO, and **(E)** LVEDV. LV EF, left ventricular ejection fraction; LV GCS, left ventricular global circumferential strain; LV GLS, left ventricular global longitudinal strain; LV GRS, left ventricular global radial strain; LV EDV, left ventricular end-diastolic volume; LGE, late gadolinium enhancement; CO, cardiac output.

There was a significant reduction in the infarct size, measured by LGE mass percentage of the left ventricular muscle mass (LVM), between the initial and follow-up CMR (19.7%, IQR 12.2–23.9 vs. 17.1%, IQR 8.3–22.5, *p* = 0.04). Initial strain parameters (GLS, GRS, and GCS) were strongly correlated with the initial edema mass and change in LGE mass between the initial and follow-up CMR. The correlations between CMR-FT parameters and CMR markers (change in LGE, edema mass, MSI, LV EF, and LV EDV) are shown in Figure 3.

**Figure 4:**
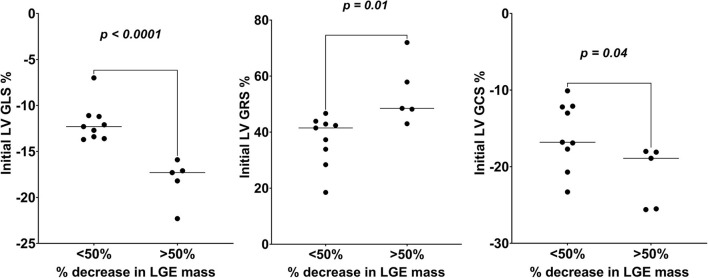
Comparison of initial LV global strains (GLS, GRS, and GCS) values of those who had >50 vs. <50% decrease in LGE mass over the follow-up study period. LV GCS, left ventricular global circumferential strain; LV GLS, left ventricular global longitudinal strain; LV GRS, left ventricular global radial strain; LGE, late gadolinium enhancement.

On ROC analysis, LV GLS% had the highest AUC for the prediction of decrease in LGE mass by 50% or more (AUC = 0.97 [95% CI, 0.95–0.99]; *P* < 0.001), with an optimal cutoff of −13.7%, followed by LV GRS% (AUC = 0.96 [95% CI, 0.85–1.1]; *P* < 0.001), with an optimal cutoff of 48.2%, and LV GCS% (AUC = 0.86 [95% CI, 0.66–1.1]; *P* < 0.001), with an optimal cutoff of −17.7%. Dividing the patients into those who did or did not have a 50% decrease in LGE mass between the initial and follow-up CMR, there was a statistically significant mean difference in baseline LV GLS% (−6.3 [95% CI, −8.94 to −3.58]; *P* < 0.0001), LV GRS% (16.6 [95% CI, 4.62–28.67]; *P* =0.01), and LV GCS% (−5.4 [95% CI, −10.51 to −0.20]; *P* = 0.04) between the two groups (Figure 4). However, there was no significant mean difference in patients who did or did not have a 50% decrease in LGE mass in other baseline CMR parameters, including LV EDV (−20.2 [95% CI, −77.9 to 37.4]; *P* = 0.46), LV ESV (−31.6 [95% CI, −84.1 to 20.9]; *P* = 0.21), LV EF (19.9 [95% CI, −4.5 to 42.3]; *P* = 0.1), and Cardiac output (0.6 [95% CI, −0.7 to 1.8]; *P* = 0.33).

## Discussion

In this prospective study, we assessed the value of myocardial global strain indices as assessed by CMR-FT in STEMI patients undergoing primary PCI. The key findings can be summarized as follows: (1) there was a significant decrease in infarct size between the initial and follow-up CMR as has been established previously, (2) LV global strain measures (GLS, GRS, and GCS) correlated with the area of myocardial edema, but not with MSI, and (3) LV strain measures (GLS, GRS, and GCS) were significantly correlated with the decrease in infarct size as measured by LGE at 6-month follow-up.

Various clinical, biochemical, and radiological predictors have been associated with clinical outcomes in patients with STEMI treated with primary PCI. Most notably, the presence of cardiogenic shock, elevated cardiac biomarkers, reduced LVEF, and large infarct size. Measured LVEF, however, may be misleading due to the hyper contractility of normal myocardium and transient post-ischemic myocardial dysfunction that may recover in time. On the other hand, early measurement of LGE provides a stronger association with late systolic dysfunction and differentiates poor late outcomes (14–16).

Recently, myocardial strain analysis has been shown to be a good predictor of clinical outcomes in patients presenting with STEMI. Previous studies using 2D speckle-tracking echocardiography showed that GLS and GCS after STEMI correlate with the final myocardial infarct size (4, 17). Furthermore, on CMR-FT, GLS demonstrates a robust and independent association with major adverse cardiac events (MACE) after STEMI, providing prognostic information incremental to common clinical and CMR imaging risk factors, including LVEF and LGE (8, 10, 18).

We found that GLS, GCS, and GRS were all reflective of the extent of acute injury of LV myocardium as measured by edema. As acute myocardial injury does extend beyond the infarcted areas, this can even involve non-culprit vessel territories (19). The most ischemic layer, the sub-endocardium, includes the longitudinal myofibers. As such, it is expected that GLS would be the most affected (20). However, in this analysis, we show that all three LV global strains, which reflect the function of the three fiber layers, are equally affected. Comparing patients who had 50% or more reduction in infarct size to those who did not have that level of improvement after STEMI, LV strain parameters differentiated between these two outcomes with high accuracy. Among all parameters, GLS had the highest accuracy with an optimal cutoff value of −13.7%. This value was in line with a similar optimal cutoff value for GLS for MACE prediction, ranging from −11 to −14% (8, 10, 18).

## Conclusions

In STEMI patients, LV global strains (GLS, GRS, and GCS) measured after primary PCI were inversely correlated with myocardial edema and decreased infarct size measured by LGE, but not MSI, cardiac output, or LVEF. We contend that initial LV global strain parameters can predict the extent of myocardial recovery.

The limitations of this study are the small sample size and short longitudinal follow-up. Given the limited cohort, we may not adequately be representing all presenting STEMI patients. Therefore, no conclusion can be made based on the lack of apparent correlation between LV global strains and systolic function or cardiac output.

## Data Availability Statement

The original contributions presented in the study are included in the article/supplementary materials, further inquiries can be directed to the corresponding author/s.

## Ethics Statement

The study was approved by the Institutional Review Board of the University of Virginia (reference number 17502), the University of Florida (reference number 201701573), and the study title Fibrocytes and Myocardial Scarring after Acute Myocardial Infarction. All patients provided written informed consent before participating in the study. The patients/participants provided their written informed consent to participate in this study.

## Author Contributions

MT and MA-A conducted image analysis, interpreted the results, and contributed to the drafting of the manuscript. MA-A, DM, and EK conceived of the idea, provided oversight for the study, and interpreted the results. EJ, EK, and MS interpreted the results and critically revised the manuscript. All authors read and approved the final manuscript.

## Funding

This work was supported by the American Heart Association award number 16IRG27180006 (EK).

## Conflict of Interest

The authors declare that the research was conducted in the absence of any commercial or financial relationships that could be construed as a potential conflict of interest.

## Publisher's Note

All claims expressed in this article are solely those of the authors and do not necessarily represent those of their affiliated organizations, or those of the publisher, the editors and the reviewers. Any product that may be evaluated in this article, or claim that may be made by its manufacturer, is not guaranteed or endorsed by the publisher.
